# Feasibility and Acceptability of Human Immunodeficiency Virus Self-Testing for Men of Middle-to-Upper Socioeconomic Status in Botswana: A Pilot Study at 4 Worksites in the Financial Sector

**DOI:** 10.1093/ofid/ofad661

**Published:** 2023-12-21

**Authors:** Keonayang Kgotlaetsile, Laura M Bogart, Nthabiseng Phaladze, David J Klein, Mosepele Mosepele

**Affiliations:** University of Botswana, Faculty of Medicine, & Boitekanelo College, Counselling Department, Gaborone, Botswana; RAND Corporation, Santa Monica, California, USA; Department of Psychiatry, Charles R. Drew University of Medicine and Science, Los Angeles, CA, USA; Faculty of Health Sciences, University of Botswana, Gaborone, Botswana; Faculty of Medicine, University of Botswana and Botswana Harvard AIDS Institute Partnership, Gaborone, Botswana; Faculty of Medicine, University of Botswana and Botswana Harvard AIDS Institute Partnership, Gaborone, Botswana

**Keywords:** Botswana, HIV self-testing (HIVST), men, stigma, sub-Saharan Africa

## Abstract

**Background:**

Although Botswana has made great progress in expanding human immunodeficiency virus (HIV) testing, men are less likely to be tested for HIV and test at a later stage compared with women. For Botswana to increase HIV testing coverage among men, HIV self-testing (HIVST) may be a promising supplement to standard, healthcare facility-based HIV testing. We conducted a pilot test of the feasibility and acceptability of HIVST for men of middle-to-upper socioeconomic status in Botswana.

**Methods:**

Thirty-five men were recruited through 4 workplaces (banking sector). Wellness officers emailed all potentially eligible male employees about the opportunity to participate. Men were surveyed at baseline and follow-up on basic sociodemographic characteristics, HIV testing history, HIV stigma, use of the HIVST kit (at follow-up), and confirmatory testing and linkage to care if a preliminary positive result is obtained (at follow-up).

**Results:**

All 35 men used the kit. The proportion who agreed with the statement that getting tested for HIV helps people feel better increased significantly from 80.7% at baseline to 100% at follow-up. In open-ended questions, men described the advantages of HIVST, including improved privacy and convenience, lowered HIV stigma, and enhanced control over testing. Concerns about HIVST included potential negative mental health consequences owing to not receiving pretest and posttest counseling, and not linking to care after a reactive result.

**Conclusions:**

Results suggest that an intervention in which HIVST is discrete, private, and under men’s control can help overcome stigma around HIV testing, resulting in a greater number of men tested.

Botswana has a high human immunodeficiency virus (HIV) incidence rate of 0.2% and high prevalence (20.8%) among those aged 15–64 years, despite great strides toward reaching the UNAIDS goal to end the HIV epidemic by 2030 [1]. In Botswana and sub-Saharan Africa (SSA) as a whole, men are less likely than women to be tested for HIV, present for HIV testing at a later disease stage [2], and experience higher AIDS-related mortality rates [3]. The most crucial and initial stage in HIV care and treatment is becoming aware of one’s HIV status. Individuals who test at a later stage cannot benefit from early antiretroviral therapy and thus have short survival times and a higher likelihood of transmitting HIV to partners [4]. Only 42% of men are estimated to be reached by healthcare facility-based testing across SSA [5]. In a 2015 study, an estimated 50% of men versus 14% of women were missed by home-based testing in Botswana [6]. Although there is free and universal healthcare in Botswana, and nearly all members of the population have close access to HIV services, including HIV testing, regardless of geography, in public healthcare facilities, men are less likely to be tested for HIV regularly and in public clinics. Men and young people in SSA contribute disproportionately to the number of people living with HIV who are not aware of their status [7].

Consistent with the theory of triadic influence [8], individual, social, and structural barriers impede HIV testing among men. HIV stigma—including internalized stigma (at the individual level) and anticipated stigma if one were to test HIV positive (at the social level)—is a key barrier to HIV testing in SSA [9–13]. Higher levels of HIV-associated stigma are associated with lower testing likelihood, especially among men [14–16]. In accordance with gender role strain and hegemonic masculinity theories [17, 18], qualitatively, men say that they worry that others will discover their serostatus if they test HIV positive, subjecting them to gossip, rejection, and shame—and loss of respect by their partner and others [9, 19–22]. Moreover, men discuss being seen accessing healthcare (and testing) [23–25], as well as an HIV diagnosis [26, 27], as a threat to masculinity—being stigmatized as weak and unable to provide for their family—with consequent loss of respect and status. Men have also said that an HIV diagnosis threatens their “respectability, independence and emotional stability” [28], their need to provide for others, and their ability to maintain a source of income [28] and higher social class [29]. HIV misconceptions among men in SSA, such as that HIV is a “death sentence,” increase stigma [9, 30, 31].

Fears about stigma and other individual-level concerns are heightened by structural factors related to healthcare, including concerns about privacy in public healthcare facilities and fears that providers will not keep their results confidential and that people from their community will see them visiting a facility for testing. Such concerns may be heightened among men of higher socioeconomic status (SES), who may fear losing status and believe that they are more recognizable in public settings [23]. Owing to inconvenient clinic hours and locations, competing needs for work are also a barrier to testing [20, 32]. Men perceive public healthcare facilities as unwelcoming and feel that seeking care is more acceptable for women [19, 24, 25, 31, 33, 34]. In addition, men may not perceive themselves to be at risk, as prevention campaigns do not target them in particular, and women are more likely to be offered testing owing to their higher level of healthcare utilization (eg, for reproductive health) [3]. Often, HIV testing strategies are not developed with men’s input and consideration of drivers and barriers to men’s health-seeking behavior (such as internalized stigma and masculinity norms). It is therefore not surprising that, despite decades of conducting multiple HIV testing strategies, HIV testing uptake among men in SSA remains at 50% [6].

HIV self-testing (HIVST) has the potential to address barriers to HIV testing among men, including HIV stigma, and thus may help achieve universal coverage of HIV testing for the general populace in SSA [35]. Men may not engage in facility-based care owing to a myriad of barriers (eg, competing needs for work, health systems that do not cater to their preferences); thus, HIVST has been proposed to be an effective strategy to reach populations regarded as “hard to reach,” including men [36]. HIVST has been shown to increase testing uptake among men who have sex with men without negative effects on linkage to HIV care [37]. HIVST may expand access to and interest in HIV testing among those not testing regularly under traditional HIV testing programs [38–40]. Integrating HIVST into comprehensive healthcare strategies can significantly contribute to the overall goal of HIV prevention, treatment, and care for men [41].

Because men in SSA are less likely than are women to be tested for HIV and also are less likely to visit healthcare providers [42], we conducted a pilot test to explore the acceptability and feasibility of implementing HIVST through a nonclinical setting—worksites—among men of middle to higher income in Botswana. We focused specifically on reaching men of middle-to-higher income because research suggests that men with more socioeconomic resources may have funds to support multiple concurrent sexual partnerships [43, 44], and middle-to-higher income men have been found to have high healthcare-related HIV stigma [31, 45]. Thus, nonclinic options, such as HIVST, may be more acceptable to men than facility-based testing and may lead to a more men being aware of their serostatus. Although HIVST has been validated as a screening tool for HIV compared with healthcare provider testing [37], research in SSA has been limited regarding nonclinical settings in which HIVST can be provided and used, where men work and congregate. Based on lessons learned from the pilot study, future research could test the effectiveness and implementation of HIVST in a range of worksites, such as major government departments and private companies.

## METHODS

### Recruitment, Screening, and Data Collection Procedures

Men were recruited through 4 worksites in the financial sector. Worksite selection was made based on reports from wellness officers of low HIV testing rates among men in higher-ranking job categories at these and other comparable workplaces. Wellness officers emailed all potentially eligible male employees about the opportunity to participate in the study. Wellness officers are professionals hired at worksites to coordinate workplace health services for employees. They interact with company staff to address their needs, at an individual level and collectively for the organization, thereby managing health risks to the worksite. The study principal investigator (PI; a medical doctor and public health researcher) made presentations at each worksite to describe the study to potentially eligible and interested employees.

The use of English was appropriate and inclusive for these men, given their high levels of education (university graduates) and the use of English as a primary language in their workplaces. In addition, when we engaged the same group of men [45], they participated in asynchronous online surveys in English. Furthermore, the use of English in surveys and presentations assisted in facilitating clear communication, enhancing data consistency. Men had the option to call the study telephone number or study email to arrange a time and place to get the test kit if they wanted to participate. Men who called the study number were screened for eligibility and, if they were eligible, they consented and were given a brief baseline survey. Men were eligible if they were ≥35 years of age, were currently employed, and had an annual income of at least 200 000 pula (about $17 000). In the last quarter of 2021, the average earnings among men in the finance and insurance sector were 17 440 pula (about $1319) [46].

A study coordinator conducted screening and surveyed men over the phone using Research Electronic Data Capture (REDCap; secure password-protected online survey software). Most men preferred to complete the screening, informed consent, and baseline survey over the phone during the same study appointment. During verbal informed consent, men were told that HIVST is only a screening test and that they would need to be given a confirmatory test by a trained healthcare provider if they tested preliminary positive or negative with HIVST. At the time of consent, men were provided with the contact information of the PI if they had questions about HIVST. They were also given contact information for other healthcare providers and counselors who could answer their questions about HIVST as well as provide confirmatory HIV testing and care (if needed) and crisis counseling. Men were provided with various options for support and confirmatory testing after an HIV-positive result. We assisted in linking them to preferred options, such as through employer-funded health coverage or publicly funded programs, private HIV care (confidential from the employer), or any option approved by the Ministry of Health and Wellness and the Botswana Health Professions Council.

Men were surveyed at baseline and once at follow-up (3–30 days after baseline), as described below. The use of English in surveys assisted in facilitating clear communication and enhancing data consistency [47]. After completing the baseline survey, men were given a choice of how to get the HIVST kit: through direct pickup from study staff at an agreed-upon location (such as at the study offices at the university); through pickup at a private, confidential space, such as self-test kits left in the wellness office at their workplace; or through delivery (such as to men’s homes or workplaces). The test kit included instructions for use of the kit in English. All test kits were labeled with a unique identification (ID) number. Men were given the option of taking a picture of the test kit (and the results, if they used the kit) and sending the picture to the study cell phone via WhatsApp (which has end-to-end encryption). They were asked to show in the photo the used or unused testing swab and their study ID label. The OraQuick HIVST used in the study has very high sensitivity and specificity [48]. Men were provided with healthcare and mental health referrals on a slip of paper in the test kit (as well as during consent).

Participants were provided with 200 pula total (about $17), in the form of 50 pula (about $3.50) each time that they completed 1 of the following 4 study activities: (1) the first study appointment, which included informed consent and a brief survey, as well as instructions about how to obtain the HIVST kit; (2) obtaining the HIVST kit (whether or not it was used); (3) returning or sending a photo of the used or unused HIVST kit; and (4) completing the follow-up survey. By incentivizing the return of unused HIVST kits, we aimed to convey to participants the acceptability of not using the kit, to encourage honest responses about lack of test kit use. The study was conducted from 5 October 2020 through 12 May 2021, and all study procedures were approved by the institutional review boards of the University of Botswana (reference UBR/RES/IRB/BIO/120), the Botswana Ministry of Health (reference HPDME/13/18/1 V1 [31]), and the RAND Corporation (ID no. 2018-0482-CR03). All study procedures were carried out in accordance with regulatory policies and guidelines. Informed consent was obtained from all participants.

### Survey Assessment

The baseline survey assessed basic sociodemographic characteristics (age, sex, marital status, highest education level, last month’s income) and prior HIV testing experiences (ever tested for HIV and if so, when and where tested). Internalized HIV stigma was assessed with the AIDS-Related Stigma Scale (developed in South Africa) [49], with items modified to be conditional (eg, “If I had HIV, I would feel dirty”), using response options from 1 (strongly disagree) to 5 (strongly agree) (α = .70). Anticipated HIV stigma if one were to test positive (eg, being disowned or neglected by family; α = .80) and stigmatizing beliefs about HIV (eg, if a member of your family became infected with HIV, would you want it to remain secret?), both with yes/no response options, were assessed with scales developed in Botswana [15, 50]. HIV testing attitudes were assessed with 2 items developed in South Africa (“Getting tested for HIV helps people feel better” and “People who test HIV positive should hide it from others”; responses ranged from 1 [strongly disagree] to 5 [strongly agree]) [51].

The follow-up survey occurred 3 days after the baseline for participants who had obtained and used the test kit. If they had not yet obtained the kit, or had obtained but not yet used the kit, they were called up to 4 more times (at 7, 14, 21, and 30 days after baseline) and asked why they did not yet obtain or use the test kit. For those who had obtained and used the test kit, the survey included items on HIV testing experiences, use of the HIVST kit, confirmatory testing, and linkage to HIV care (if a preliminary positive result was obtained), HIV stigma, anticipated HIV stigma, and attitudes toward HIV testing. Participants were asked open-ended questions about their experience with HIVST: How much was using the HIV self-testing kit a good experience or not a good experience, and why? What did you like the most about using the HIV self-testing kit? What did you like the least about using the HIV self-testing kit? The study coordinator recorded verbatim responses using REDCap. The average duration of the survey was 30–45 minutes.

### Statistical and Qualitative Analysis

We examined descriptive statistics for all study variables and conducted paired *t* tests to test differences between intentions to use HIVST, internalized and anticipated stigma, stigmatizing beliefs, and beliefs about HIV testing at baseline and follow-up. The research coordinator and the study principal investigators were actively involved in monitoring the data collection procedures and the research context throughout the investigation to encourage reflexivity. This made it possible for the interviewer to understand where she stood in relation to the participants and herself (to help guard against biases). For the qualitative data (responses to open-ended questions), we used directed content analysis, in which we allowed themes to emerge to describe reasons for liking or disliking HIVST, considering prior themes that had emerged in our formative work [45, 52]. Two female team members (a co-PI from the United States and a study coordinator from Botswana) read all open-ended responses, from which they generated a codebook listing key themes. Coding categories are shown. After achieving good consistency between coders (Cohen’s κ = 0.821 across 40 excerpts), the coordinator coded the remaining excerpts (which were reviewed by the co-PI). After coding, coders reviewed all excerpts and came to a consensus about key themes.

## RESULTS

### Baseline Participant Description

Of 46 men who were screened, 36 were eligible, of whom 35 completed the baseline survey and 32 completed the follow-up survey (see Table 1). As shown in Table 1, the participants’ average age (standard deviation) was 43.0 (5.9) years; 21 (60%) were married, and all had at least a university diploma. All had tested for HIV before the baseline survey, but only 15 (42.9%) had tested in the past year. At baseline, 28 participants (80%) said that they were very likely to use the HIVST kit in the next month.

**Table 1.** Participant Characteristics at Baseline and Follow-up Responses

Characteristic or Response	Participants, % (No.)^a^
Baseline characteristics (n = 35)	
Age, mean (SD), y	43.0 (5.9)
Married status	60.0 (21)
University diploma or higher education level	100 (35)
Ever tested	100 (35)
Tested <1 y earlier	42.9 (15)
Very/somewhat likely to use HIVST within 1 mo	80.0 (28)
Follow-up responses (n = 32)	
Received and used HIVST (delivered to workplace)	100 (32)
Storage	
Stored at home	48.4 (15)
Stored at work	29.0 (9)
Stored elsewhere (bag, car, golf club)	15.2 (5)
Did not store (used immediately)	6.1 (2)
Usage	
Used at home	68.8 (22)
Used at work	28.1 (9)
Used elsewhere (golf club private room)	3.1 (1)
Instructions “very clear”	96.9 (31)
Test kit “somewhat/very easy to use”	100 (32)
Confident that the kit was used correctly	100 (32)
Confident that the results were read correctly	96.9 (31)

Abbreviation: HIVST, human immunodeficiency virus self-testing; SD, standard deviation.

^a^Data represent % (no.) of participants unless otherwise specified.

### Stigma Perceptions

As shown in Table 2, among those the 32 participants who also completed the follow-up survey, at baseline most reported HIV discrimination concerns (eg, losing a spouse/intimate partner) 62.5% (n = 20), and 43.8% (n = 14) endorsed at least one stigmatizing belief about HIV. There was a significant increase in the percentage of participants who agreed with the statement that getting tested for HIV helps people feel better, 80.7% at baseline versus 100% at follow-up (*P* = .01). There were no significant differences between baseline and follow-up for internalized stigma, anticipated HIV stigma, any stigmatizing beliefs about HIV, and any HIV testing beliefs.

**Table 2.** Perceptions Related to Human Immunodeficiency Virus Testing and Stigma (Baseline and Follow-up; n = 32)

Perception	Participants, % (No.)^a^		McNemar χ^2^(1)^b^; *P* Value
	Baseline	Follow-up	
Internalized HIV stigma score, mean (SD)^c^	2.3 (0.9)	2.0 (1.0)	1.5 (31)^d^; .15
Any anticipated HIV stigma	62.5 (20)	68.8 (18)	0.7; .41
Any stigmatizing HIV belief	43.8 (14)	59.4 (19)	1.7; .20
Agreement that being tested for HIV helps people feel better	80.7 (25)	100 (32)	6.0; .01^e^
Agreement that people who test HIV positive should hide it from others	28.1 (9)	18.8 (6)	1.3; .26

Abbreviations: HIV, human immunodeficiency virus; SD, standard deviation.

^a^Data represent % (no.) of participants unless otherwise specified.

^b^Values in this column represent McNemar χ^2^(1) unless otherwise specified.

^c^Score based on AIDS-Related Stigma Scale [49].

^d^Paired *t* value (*df*).

^e^Significant at *P* = .05.

### HIVST Receipt and Use

All 35 men requested that HIVST be delivered to their workplace or another convenient location other than home, and all used the kit. All men who obtained the kit sent a picture of the used test kit (with the result and ID no. showing) via WhatsApp to the study cell phone. Follow-up surveys showed that men stored the kits before use at a variety of places (home, work, other), and most used them at home. All or nearly all found HIVST easy to use, were confident that they used it correctly, and said that they were likely to use it in the next year.

### Qualitative Responses About HIVST

Although the decrease in internalized stigma was not significant at follow-up, open-ended responses indicated positive attitudes about HIVST (Table 3). Men discussed the convenience of being able to test wherever they wanted, as well as the advantage of privacy, which led to reduced fear of stigma. However, men expressed concerns about confidentiality and privacy and about where the test kits should be placed for easy access and/or delivery. Some men may not have had sufficient privacy to participate in the study or to use HIVST from home.

**Table 3.** Responses to Open-Ended Questions After Use of Human Immunodeficiency Virus Self-Testing

Question	Representative Quotes
What did you like most about using the test kit?	“I liked the convenience that I can do it at my own time and the privacy that comes with it of doing it alone.” (38-y-old man) “The fact that it came in a gift bag… It did not come to me as if it's something medical, something to do with a stigma even the HIV/AIDS.” (39-y-old man) “I liked the fact that you just swab in the mouth. You know, some people are afraid of the pricking, even though I am not afraid, some are afraid, and they can be encouraged to do the test now because the pricking will not be there.” (42-y-old man)
How much was using the HIV self-testing kit a good experience or not a good experience?	“A very good experience—the instructions are very clear and there were pictures, and I was able to follow through; it was also a simple exercise to do, and it does not take too much time.” (38-y-old man) “It was very convenient compared to if I had to go to a facility to test; I did the test by myself and no one else saw my results and another person can only know if I decide to tell them.” (37-y-old man) “To me it was a good experience because I am actually in control when using it though I was a bit nervous, I thought of leaving it and doing the next day but I knew that it will be hanging in the back of my head because I won't be able to sleep if I don’t do it… I became a bit impatient and I finally decided to do it. When I opened it I was a bit nervous because there is always that ‘what if thoughts’ but it was good because it is different compared to when you go to a clinic or some other place. You find that prior to testing and when waiting for the results the communication that the personnel gives us sometimes is not good as they can give you an attitude that maybe you are already positive, they have poor communication skills because sometimes you can even think of giving up along the way. With this one you find that I do it alone without anyone having to influence me.” (47-y-old man)
What did you like the least about using the HIV self-testing kit?	“To me, it is a good method, if I had the powers, I would like for it to be available to everyone to use it.” (47-y-old man) “Not having someone to talk to and calming my emotions before doing the test, because where I usually test, I get counseling and advice before getting tested.” (51-y-old man) “In case you don't do it right you might get the wrong results and then panic.” (53-y-old man) “Not really the test in particular, but in a case where one is alone, and they do the test. Usually before testing there has to be some emphasis on precounseling that can be done. Though there was a contact slip given with the test kit some people would not call to use those because they are not free to open up so there is need for emphasis on precounseling if we are to use this.” (39-y-old man)

Abbreviation: HIV, human immunodeficiency virus.

Another issue was concern about using the test kit. Men expressed anxiety regarding their ability to conduct the test and read results, as well as the mental health effects of a positive result, even though the team provided detailed information about whom to call and what to do. Fears about mental health effects were amplified by stigma (not wanting others to know about a positive result but fearing that their reaction to a positive result would lead others to figure out their serostatus).

## DISCUSSION

In the present study, men discussed the benefits of HIVST, as being simple and convenient, and they valued the privacy and autonomy of the testing process (ie, control over where and when they are tested and how and when their results are disclosed), which reduced fears related to HIV stigma and empowered them to test more in the future. Men stored the kits in a variety of places (home, work, or other), but most used them at home. The findings of the current study on the feasibility and acceptability of HIVST among men of middle to upper SES in Botswana are consistent with the larger literature indicating high acceptability and feasibility of HIVST among key and other populations [9, 38, 53], as well as studies in settings with limited resources showing that men opt to use HIV testing provided outside a clinic environment [9, 54, 55].

In contrast to other studies that have been found in the literature, this study took sex and socioeconomic class into account, focusing on the unique needs of men of middle to high SES in Botswana. Specifically, we met men where they lived and/or worked to provide HIV testing, by recruiting them through their worksites and giving them the opportunity to access HIV testing outside a clinical environment, at a place of their choosing. Extending earlier studies indicating stigma and masculinity-related issues regarding HIV testing [24, 25], the study results contribute to the understanding of why men of higher SES may be more reluctant to be tested. We discovered that it is crucial to further explore stigma-related concerns to determine how to increase testing among men. HIVST among men plays a pivotal role in promoting access to testing, reducing stigma, and ultimately enhancing the overall health outcomes of specific groups affected by HIV [56, 57].

HIVST has been shown to lower social and structural barriers connected to facility-based and community-based testing and generate demand among those unreached by clinic-based testing services by enabling individuals to perform an HIV test privately and conveniently at home [16, 58–61]. Similarly, in our study men discussed the convenience of being able to test wherever they wanted, as well as privacy, which led to reduced fear of stigma [58, 62]. Nonetheless, men expressed concerns about confidentiality and privacy around the use, access, and delivery of the test kit. Some men may not have had the privacy to participate in the study or to use HIVST kits from home. Future work could determine where best to place HIVST kits for easy access, without compromising privacy and confidentiality, for men interested in using HIVST.

This study discovered issues regarding the requirement for HIVST counseling, as did prior studies [9]. Men showed concern about their ability to perform the test and understand both the outcomes and the possible effects of a positive result on their mental health. Some barriers included fears about mental health effects amplified by stigmatization (not wanting others to know about a positive result but worried that their reaction to a positive result would cause others to figure out their serostatus), exacerbating concerns about the effects on mental health [45]. Our results suggest the importance of providing healthcare referrals and mental health counseling to men if needed in a way that is acceptable and, if possible, outside physical healthcare facilities (eg, via phone or telehealth).

Our study’s strengths lie in the mixed-methods approach to understanding HIVST acceptability and feasibility among men, as well as our focus on an understudied population of men at risk of HIV infection. Although the present study extends the literature by showing that HIVST can address men’s barriers to HIV testing, study limitations should be acknowledged. The sample size was small, consistent with the goal of this pilot study to determine acceptability and feasibility of HIVST for men recruited through worksites, rather than testing the effectiveness of our worksite implementation strategy in increasing the proportion of men at worksites who are aware of their HIV-positive serostatus. The sample size was too low to examine statistically significant differences but sufficient for initial data on feasibility and acceptability. We were unable to fully evaluate our pilot intervention owing to coronavirus disease 2019 (COVID-19); compared with what was originally planned, the sample size was lower, owing to difficulties with recruitment.

The unique demographics and contextual differences that these men presented with, compared with other jurisdictions, could also have contributed to the lower sample size. Despite this, the smaller sample size was sufficient to capture the specific nuances of the target population. COVID-19 orders included working from home and restricted in-country movement; thus, our intended study procedures, which included recruitment via worksites, in person, and delivering or picking up test kits, were not possible to carry out during much of the pandemic. Moreover, men may not have been able to use the test kit in privacy at home, and they were restricted in the places they could go to use the test kit away from home during the pandemic.

Finally, participants were self-selected into the study and used HIVST, which limits the generalizability of the results. Although the sample size was small, invitations were extended across 4 worksites to obtain a range of men for whom HIVST acceptability and feasibility could be evaluated. Participants who did not use HIVST at follow-up continued to be called, which could have increased the likelihood that they eventually used HIVST.

In conclusion, the present results show that men who use HIVST view it as an acceptable and feasible option for being tested outside of clinical settings. However, offering men a variety of options to obtain HIVST outside their worksites may be necessary to engage many men to use HIVST. To reach men who are uninformed about HIVST, community-based models may be required, such as promoting and offering HIVST in settings that men frequent or where they congregate, including places of business (eg, pharmacies) or community events (eg, health fairs). Overall, offering HIV testing outside of a clinical setting may lessen barriers related to HIV stigma, privacy, and time constraints.
